# Identification methods as a factor affecting the performance of clinical microbiology laboratories participating in an external quality assessment program: a cross-sectional, retrospective analysis

**DOI:** 10.1099/jmm.0.001915

**Published:** 2024-10-29

**Authors:** Jennifer Wu, Md Saiful Alam, Veronica Restelli, Selvarani Vimalanathan, Lucy A. Perrone

**Affiliations:** 1 Canadian Microbiology Proficiency Testing Program (CMPT), Department of Pathology and Laboratory Medicine, The University of British Columbia, Vancouver, British Columbia, Canada; 2 School of Population and Public Health, The University of British Columbia, Vancouver, British Columbia, Canada

**Keywords:** clinical microbiology laboratory, diagnostic testing, proficiency testing, quality assurance

## Abstract

**Introduction.** Laboratory participation in external quality assessment (EQA) programmes including proficiency testing (PT) is a requirement of clinical laboratory conformance to ISO 15189:2022 *Medical laboratories – Requirements for quality and competence*. PT is one EQA method whereby laboratories are sent blinded samples for characterization by routine laboratory diagnostic methods. Importantly, PT enables a laboratory’s performance to be evaluated in comparison to the standard reference methods and to the performance of other peer laboratories using similar diagnostic methods.

**Gap statement.** The desired outcome of participating in PT is to help laboratories identify possible sources of error in each step of the total testing process and particularly in their test methods during the analytical phase.

**Aim.** This cross-sectional study investigated the impact of using matrix-assisted laser desorption ionization time-of-flight mass spectrometry (MALDI-TOF MS) compared to conventional phenotypic biochemical testing on laboratory performance in a clinical bacteriology PT scheme.

**Methodology.** During a 6-year period from 2017-2022, the Canadian Microbiology Proficiency Testing implemented 112 PT challenges comprising 22 different sample types and included 61 different bacterial species. This was translated into 5883 graded test events for analysis. Multiple logistic regression techniques were employed to explore the association between the test method employed and laboratory performance. The sample type and aerobic classification of challenge organisms were included as confounding variables.

**Results.** Laboratories using MALDI-TOF MS performed significantly better in characterizing microorganisms than laboratories using phenotypic biochemical testing alone [odds ratio OR = 5.68, confidence interval (CI): 3.92, 8.22] regardless of the sample type and aerobic classification. Notably, our analysis identified a significant association between anaerobic organisms and laboratory performance (OR: 0.24, CI: 0.17–0.35), suggesting that culturing and identifying fastidious organisms remains a significant obstacle for many clinical microbiology laboratories.

**Conclusions.** Although no method is infallible and its performance will depend on the validation and quality assurance procedures, this finding may help the management in the decision for implementing MALDI-TOF MS in the microbiology laboratory. This study highlights the important role PT providers play in the objective assessment of laboratory performance and how it can provide evidence for quality improvement.

## Introduction

Clinical microbiology laboratories are essential for effective patient care because they provide crucial information regarding the characterization of infectious pathogens and inform effective pharmaceutical treatment. They confirm infectious disease outbreaks and routinely monitor the development and spread of antimicrobial resistance in communities. They provide critical data for health policy development and inform public health programming. Errors in the clinical laboratory put the safety of the patient and community at risk. Considering laboratory data influence up to 80% of decisions in health care and given the large number of laboratory tests performed, even low rates of laboratory error may reflect a significant number of patients affected [1]. The recognition of quality failures in the total testing process is important as it helps identify weaknesses in the system [2]. If incidents are not systematically recognized and analysed, any quality improvement or reduction in the risk of recurrence is unlikely [3].

Proficiency testing (PT) is an external quality assessment (EQA) method in which laboratories are sent simulated clinical samples or bacterial isolates for testing by routine laboratory methods. PT also allows a clinical laboratory’s performance to be assessed in comparison to the reference methods and to other peer laboratories [4]. In addition, there is evidence that suggests improved practices by laboratories can be associated with performance of PT over long periods of time [5,6]. PT programmes are an integral element of strong laboratory and health systems because they provide an objective service of assessment in accordance with the international standards of quality.

PT identifies sources of error from pre- to post-analysis, including misidentification, misinterpretation of normal flora and inappropriate reporting. Many bacteriology PT programmes send samples (also commonly referred to as PT challenges) as lyophilized cultures and address only the examination phase of the total laboratory testing process [7,8]. A variation of traditional PT is the creation and dissemination of clinical relevancy challenges, which look like and act like clinical samples [9]. Rather than only focusing on the traditional analytic phase of the laboratory cycle, clinical relevancy challenges also address pre- and post-analytic components of the laboratory cycle, including interpreting test results and creating reports. The product of such combined and integrated efforts results in clinically relevant microbiology [10,11].

Among one of the many factors that may influence a laboratory’s ability to report accurate results is the diagnostic method used to identify pathogens. Useful laboratory information in microbiology must provide more than just an accurate assessment of present flora. Specimens must be processed in a timely fashion and on appropriate media to optimize finding critical pathogens, pathogens need to be recognized and be differentiated from normal flora, information needs to be interpreted in a clinically meaningful manner and antibiotic susceptibility testing (AST) must be performed using clinically appropriate drug selections. For example, 16S rRNA gene sequencing is becoming widely accepted as the new standard for microbial identification and diagnosis [12,13]. This sequencing method allows microbiologists to achieve genus-level sensitivity for metagenomic surveys of mixed bacterial populations, but it is predominately used by larger reference clinical laboratories due to the significant costs involved. Phenotypic identification by means of interpreting morphological and biochemical characteristics has long been the conventional method used by clinical microbiology laboratories for the routine identification of bacteria. The samples are subcultured for morphological analysis and subjected to a series of biochemical tests either manually or using an automated system such as VITEK 2 (Biomerieux), BD Phoenix (Becton Dickinson) and Microscan (Beckman Coulter). Despite its prevalence, the studies have demonstrated that phenotypic testing as an identification method is not ideal for fastidious and/or biochemically non-reactive organisms [14].

A growing number of laboratories have integrated matrix-assisted laser desorption ionization time-of-flight mass spectrometry (MALDI-TOF MS) into their workflow to replace or complement phenotypic testing as a culture-independent method [15]. MALDI-TOF MS utilizes laser technology and time-of-flight mass analysers to separate analytes within the protein extract of the samples by their mass-to-charge ratio (*m*/*z*). The corresponding spectra of the unknown organism are then matched against a database containing spectral profiles of known bacterial isolates for identification. Morphologically or biochemical similar pathogens can be speciated based on their *m*/*z* spectra when phenotypic testing is inconclusive. MALDI-TOF MS is also highly sensitive, requiring only a small amount of microbial biomass to generate a spectral profile, therefore eliminating the need for time-consuming subcultures. All in all, implementing MALDI-TOF MS can expedite microbial identification results and antimicrobial treatment [13].

In this cross-sectional study, the results reported by laboratories participating in the Canadian Microbiology Proficiency Testing (CMPT)’s clinical bacteriology PT programme from 2017 to 2022 were analysed to investigate the association between laboratory performance and identification method, with MALDI-TOF MS and phenotypic biochemical testing as the two main identification methods. Sample site sterility and organism respiration were also included in the analysis as relevant confounding variables.

## Methods

### The clinical bacteriology PT scheme

The CMPT is accredited as an EQA provider to ISO 17043 : 2022 *Conformity assessment – General requirements for the competence of proficiency testing providers* by A2LA. The CMPT’s clinical bacteriology scheme is consisted of four PT surveys distributed to the participating laboratories over a period of 12 months in each programme year. Each survey consisted of between six and nine different challenges (each are individual samples). Each challenge/sample included in the survey was accompanied by a patient case history, description of specimen source (e.g. wound) and the expected antibiotic susceptibility if required. Sample challenges including the selection of micro-organisms were informed by expert technical committee, in consideration of previous challenge rounds and results, emerging pathogens, changing diagnostic methods, changes to testing guidelines, and other contemporary factors. The testing event ‘window’ was 14 days between sample shipping and result submission to the secure CMPT members online portal.

### Sample production

PT samples were manufactured in the CMPT’s ISO 9001 : 2005 certified laboratory. The samples were developed following the proprietary CMPT protocols to simulate patient samples and were often liquid and wet-mounted slides – not lyophilized. Sample composition, aspect and behaviour were tested and quality was confirmed by reference laboratories before they were sent to the participants. Sample formulations were tested for homogeneity, commutability and stability up to 21 days post-production at room temperature. PT samples were labelled with production date, sample source and a sample identification number. PT sample composition including organisms included in the challenge was unknown to the participants. PT samples were shipped to participant laboratories with detailed processing and reporting instructions. The laboratories were instructed to use routine testing protocols and process the PT samples as they do patient samples.

### Evaluation and grading

The CMPT uses the established grading guidelines and a consensus-based process of peer evaluation [16]. All participant results for each test event were entered in the CMPT’s members online portal [17], which is secured by unique user log-in firewalls and accessible only to programme participants. Each participant was asked to include the following information: date of sample receipt, date of testing, reporting person, testing methods and results. The CMPT does not collect detailed information about the laboratory facilities. After the 14-day survey deadline, participant results were analysed and graded for each challenge round. Participant result reports must include accurate information that is sufficient for interpretation and appropriate clinical decision-making. Each challenge round included different variables that can be graded separately, e.g. identification of organism and susceptibility to a specific antibiotic. All grades need to meet the consensus criteria before being considered appropriate for assessment. For the clinical bacteriology programme, a challenge is considered suitable for grading if agreement is reached by 80% of the selected reference laboratories and at least 50% of the participants. Participant result reports that contain errant information or are missing clinically important information are graded ‘unacceptable’. The deidentified, aggregated data were summarized and uploaded to the CMPT’s members portal to allow for valuable inter-laboratory comparison of the results.

### Data selection and cleaning

The test results submitted between 2017 and 2022 by participants in the CMPT’s clinical bacteriology PT programme were identified through the CMPT’s database. All the participants reported the organisms they identified and the identification method used for each challenge. AST results were also submitted, but not analysed in this particular study.

Participant performance data were organized into individual profiles and allowed for our study of laboratory performance according to identification method, sample sterility, mode of respiration and year. Methods used by laboratories to identify organisms were sorted by (1) the methods employed that relied on the phenotypic identification of organisms through standard biochemical testing (e.g. VITEK) and (2) the methods employed that relied on the molecular identification of organisms (e.g. MALDI-TOF MS and RNA sequencing techniques). The sterility and mode of respiration used by the organisms present in each challenge were defined by the CMPT according to the sample type and organism, respectively. Challenges simulating samples originating from sterile sites such as blood, peritoneal fluid, cerebrospinal fluid, abscess, joint fluid, bone, tissue, intraocular fluid and urine were classified as sterile, while challenges simulating samples originating from non-sterile sites such as stool, wound, cervix, sputum, throat, ear, eye, urethra, vaginal, oesophagus and groin sample types were classified as non-sterile. The mode of respiration used by each organism was designated as either aerobic or anaerobic according to their culture conditions. Facultative anaerobic organisms capable of utilizing both aerobic and anaerobic respirations were classified as aerobes, as they would be cultured under aerobic conditions. Similarly, challenges containing multiple organisms constituting normal flora were also classified as aerobic.

### Statistical analysis

**Fig. 1.**
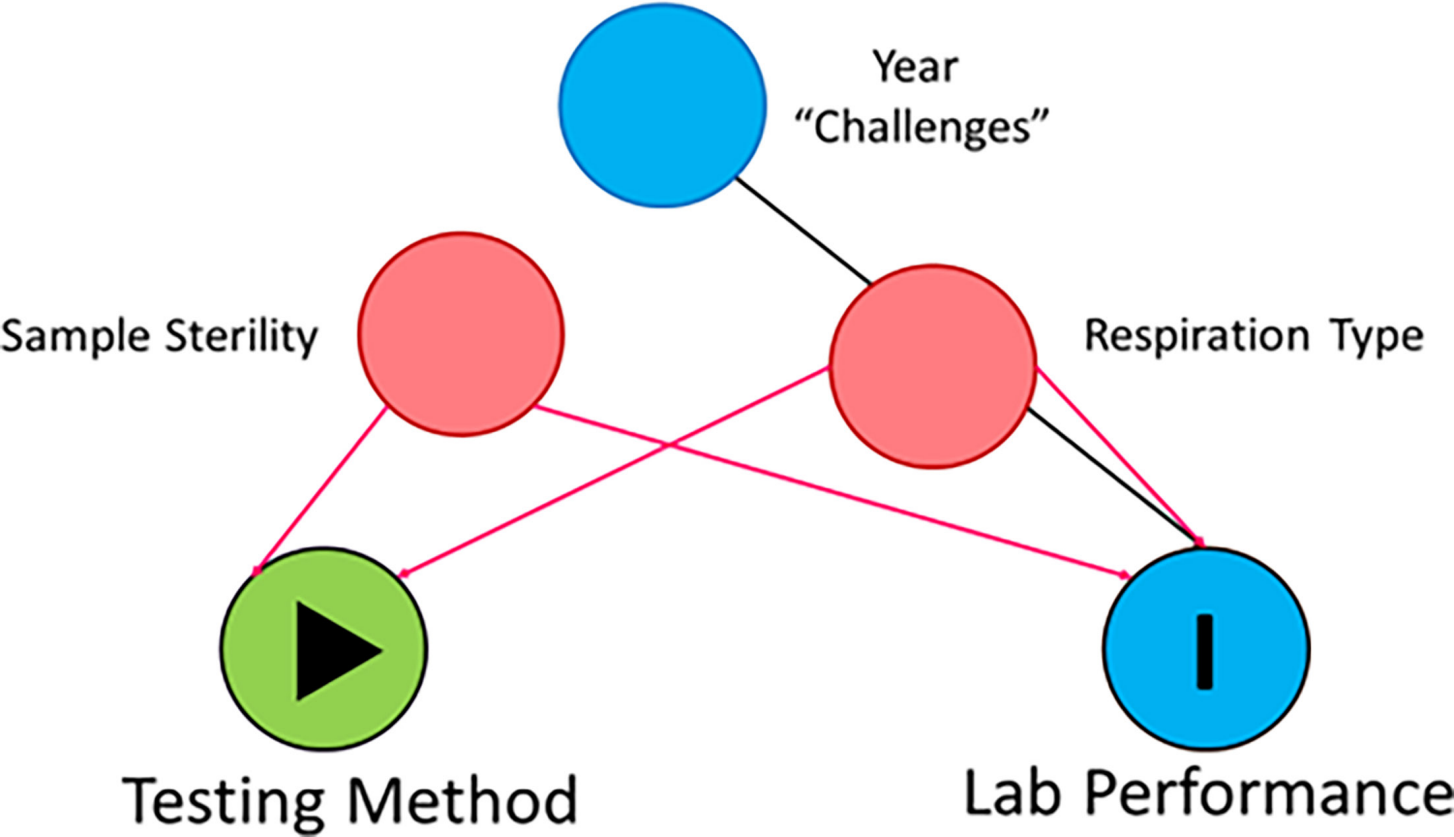
A directed acyclic graph (DAG) model illustrating the minimal sufficient confounding adjustment sets in the relationship between the testing method and laboratory performance among participants.

Statistical analysis was conducted using STATA version 17 and R version 4.2.1. The chi-square test was utilized to compare the distribution of each variable concerning the outcome variable ‘laboratory performance’, expressed as frequencies and percentages. Multiple logistic regression techniques were employed for a complete case analysis to explore the association between the testing method and laboratory performance. Relevant confounders (sample site sterility and respiration type), as well as risk factors for the outcome (year: challenges), were identified based on the existing literature and conceptualized using a directed acyclic graph (DAG) model (Fig. 1). The DAG model was adjusted for the minimal sufficient adjustment set derived from the DAG, which included sample sterility and respiration type. The risk factor for the outcome, such as year: challenges, was included in the automated backward stepwise regression with the Akaike information criterion [18] to enhance the estimated precision. The upper bound of the model incorporated both the minimal sufficient adjustment set and the risk factors for the outcome, while the lower bound included only the minimal sufficient adjustment set. We also tested for the interaction effect of sample sterility in relation to exposure and outcome. Once the main effect model was defined, ANOVA was used to assess whether the model that included the interaction terms was statistically significant. The model performance was assessed using a receiver operating characteristic curve, and goodness of fit was determined using the Hosmer–Lemeshow test. Measures of association were reported using both odds ratio (OR) and confidence interval (CI). All statistical tests were two sided, with a significance level set at *P* < 0.05.

## Results

During the period from 2017 to 2022, 120 individual challenges were sent to 74 individual subscribing laboratories across Canada. The majority of participating laboratories were hospital based (86.5%). This study included only five independent outpatient laboratories and five public health laboratories. A total of 120 challenges were sent during the study period; 9 challenges were ungraded, leaving 111 challenges for detailed analysis. 32.5% of the challenges were from sterile sites and 67.5% were sampled from non-sterile sites (Table 1).

**Table 1.** Sample type and frequency sent to participant laboratories (2017–2022)

Sample site status	Sample type (no. of challenges)
Sterile (*n* = 39)	Abscess (3)
	Blood (14)
	Bone (2)
	CSF (4)
	Intraocular fluid (1)
	Joint fluid (6)
	Peritoneal fluid (3)
	Tissue (4)
Non-sterile (*n* = 81)	Cervix (1)
	Ear (3)
	Oesophagus (1)
	Eye (3)
	Groin (1)
	Sputum (8)
	Stool (9)
	Throat (9)
	Urethra (1)
	Urine (15)
	Vaginal (3)
	Wound (22)
Total	112

A total of 5883 testing events (results) were graded and analysed. Of the 5883 test events, 5602 (95.22%) were considered acceptable results and 281 (4.78%) were considered unacceptable (Table 2). Graded testing events were also analysed by type of sample (e.g. site sterility), year of testing, method used for organism identification and type of target organism (e.g. respiration type).

**Table 2.** Baseline characteristics of laboratories participating in the programme (2017–2022)

Parameters	Frequency (percentages)
Average participating labs, n = 74	
Total number of challenges sent: 120 (111 had graded components, 9 were completely ungraded)	
Total number of graded test events	5883
Laboratory performance	
Unacceptable grades	281
Acceptable grades	5602
Testing methods	
Phenotypic	3499
Mass spectrometry (MS)	2384
Sterility status	
Non-sterile	4009
Sterile	1873
Programme year	
2017	1065
2018	928
2019	961
2020	1066
2021	928
2022	935
Respiration type	
Aerobic	5199
Anaerobic	683

Laboratory performance was classified as either unacceptable or acceptable (Table 3). For testing methods, 6.95% (243/3498) of responses using phenotypic methods were unacceptable, compared to only 1.34% (32/2384) unacceptable with MALDI-TOF MS (*P* < 0.001). This suggests a significant association between the testing method and laboratory performance. For organism type, unacceptable performance was 11.86% (81/683) for anaerobic organisms compared to 3.79% (191/5043) for aerobic organisms. This indicates a significant association between organism type and laboratory performances. No significant difference was observed regarding site sterility status, with 4.72% (154/3266) unacceptable performance for samples from non-sterile sites and 4.63% (121/2616) for samples from sterile sites (*P* > 0.05). Broadly, unacceptable performance ranged from 4.79% (51/1065) in 2017 to 5.13% (48/935) in 2022, with no clear pattern across years.

**Table 3.** Bivariate analysis of laboratories participating in the PT programme from 2017 to 2022 stratified by unacceptable/acceptable laboratory performance

Parameters	Laboratory performance	
	**Unacceptable no. of responses**	**Acceptable no. of responses**
Primary exposure		
Testing methods**		
Phenotypic	244 (7.01%)	3238 (93.00%)
MALDI- TOF MS	30 (1.25%)	2370 (98.75%)
Confounders		
Site sterility status		
Non-sterile	182 (4.54%)	3827 (95.46%)
Sterile	92 (4.91%)	1781 (95.09%)
Year challenge was sent out*		
2017	51 (4.79%)	1014 (95.21%)
2018	18 (1.94%)	910 (98.06%)
2019	44 (4.58%)	9156 (95.42%)
2020	59 (5.53%)	1007 (94.47%)
2021	55 (5.93%)	873 (94.07%)
2022	48 (5.13%)	887 (94.87%)
Organism type**		
Aerobic	193 (3.79%)	5006 (96.21%)
Anaerobic	81 (11.86%)	602 (88.14%)

* <0.05,*P < 0.01, **P < 0.001.

The results of crude and adjusted logistic regression analyses evaluating the associations between testing methods and laboratory performance among laboratories participating in the programme are shown in Table 4. The outcome was unacceptable (reference category) vs. acceptable laboratory performance. In the unadjusted analysis, mass spectrometry (MS) testing was associated with 5.25 times higher odds of acceptable performance compared to biochemical methods (OR: 5.95, 95% CI: 3.65–7.54, *P* < 0.001). After adjusting for other factors, MS testing still had 5.68 times higher odds of acceptable performance (Adjusted Odds Ratio (AOR): 5.68, 95% CI: 3.92–8.22, *P* < 0.001). Anaerobic organisms had lower odds of acceptable performance compared to aerobic in crude (OR: 0.29, 95% CI: 0.19–0.38, *P* < 0.001) and adjusted analyses (AOR: 0.24, 95% CI: 0.17–0.35, *P* < 0.001). No significant associations were found between site sterility status and laboratory performance in crude (OR: 1.02, 95% CI: 0.89–1.15) or adjusted models (AOR: 1.03, 95% CI: 0.66–1.15).

**Table 4.** Crude and adjusted associations between the testing method and lab performance among laboratories participating in the PT programme from 2017 to 2022 stratified by unacceptable/acceptable laboratory performance

	Unadjusted OR (95% CI)	Adjusted OR (95% CI)
Primary exposure		
Testing methods		
Biochemical	Ref	Ref
Mass spectrometry (MS)	5.25 (3.65–7.54)*	5.68 (3.92–8.22)*
Site sterility status		
Non-sterile	Ref	Ref
Sterile	1.02 (0.89–1.15)	1.03 (0.66–1.15)
Year challenge was sent out		
2017	Ref	Ref
2018	2.41 (1.41–4.12)*	2.54 (1.46–4.42)*
2019	1.01 (0.66–1.51)	1.31 (0.86–2.01)
2020	0.86 (0.58–1.26)	0.99 (0.66–1.49)
2021	0.80 (0.54–1.18)	0.84 (0.55–1.27)
2022	0.87 (0.59–1.30)	0.61 (0.40–0.94)*
Respiration type		
Aerobic	Ref	Ref
Anaerobic	0.29 (0.19–0.38)*	0.24 (0.17–0.35)*
Microaerophile	1.47 (0.53–4.11)	1.52 (0.54–4.32)

* <0.05, , *P < 0.001.

## Discussion

Participation in PT programmes is essential for quality assurance in a medical laboratory setting. Ultimately, it is a learning exercise intended to help subscribers identify possible sources of error in every step of the total testing process. The accuracy and precision of MALDI-TOF MS in identifying organisms have been well reported in the literature and had been shown to produce more accurate results than phenotypic biochemical testing at a fraction of the time and cost [19,23]. We approached this retrospective study with a curiosity about the influence of testing methods on performance outcomes. This study presents a performance overview of clinical microbiology laboratories and demonstrates that laboratories using MALDI-TOF MS as the primary method for microbial identification were 5.68 times more likely to report an acceptable result compared to laboratories using phenotypic testing, regardless of the sample type.

While no significant association was observed between the sample origin (sterile site vs. non-sterile site) and laboratory performance, when anaerobic organisms were present in the PT sample, laboratories had lower odds of acceptable performance. Anaerobic infections are often missed by laboratories as successful isolation requires special culture and identification techniques. Anaerobes are often slow growing and yield only small colonies, and their identification is further complicated by the fact that they are often biochemically non-reactive, making it difficult to distinguish between different species [14]. The respiration type of organisms was consequently selected as a confounding variable in this study to evaluate the ability of laboratories to identify anaerobic organisms. MALDI-TOF MS has been established as an alternative to phenotypic testing for the identification of fastidious and anaerobic organisms, as a spectral fingerprint can be generated from as little biomass as a single colony. Thus, laboratories using MALDI-TOF MS are able to skip the time-consuming subculturing process and test directly from the primary culture plate [12,14,24]. The improvement in accurate anaerobe identification when using MALDI-TOF MS was demonstrated in a 2014 study by Li *et al.*, where MALDI-TOF MS correctly identified 92% of anaerobic clinical isolates at the species level, compared to the 85% identified using an automated phenotypic system [19]. This was again demonstrated by Yunoki *et al.* in a 2016 study, where MALDI-TOF MS correctly identified 80% of anaerobic clinical isolates at the species level, compared to the 58% identified using the same automated phenotypic system [22]. Additionally, both studies reported a greater number of ‘no identification’ results when using phenotypic testing. While the results of our study demonstrate that MALDI-TOF MS was associated with improved identification of anaerobes, a similar improvement was observed in aerobes. This finding emphasizes the overwhelmingly positive impact MALDI-TOF MS has on microbial identification.

Laboratories are expected to interpret the identification results within the context of the sample collection site. The samples from sterile sites can be easier to interpret as any organism growing in the sample would generally be considered significant [25]. Samples from non-sterile sites pose an additional challenge for laboratories as they need to differentiate pathogen vs. normal flora or the significance of the isolated organism [25]. Our study ultimately found no significant difference in the frequency of unacceptable results when results from sterile site PT samples were compared to results from non-sterile site samples, indicating that a laboratory’s ability to accurately identify and report bacterial pathogens is independent of the sample site type.

In addition, because a clinically relevant report should not only look at the identification of the organism but also its significance in the sample, the criteria for an acceptable or unacceptable grade will vary depending on the organism, sample type and clinical scenario for each challenge. Therefore, the inability to speciate a pathogen was not necessarily considered an unacceptable result depending on the context of the sample. This differs from previous studies focused on validating MALDI-TOF MS as an identification method, rather than the ability of laboratories to effectively use MALDI-TOF MS [19,22]. As a result, culturing errors, including contamination or no microbial growth, and inappropriate reporting were also causes for an unacceptable grade in this study. Ultimately, we found that the leading cause for an unacceptable grade was the inability to correctly identify the pathogen as required by the clinical scenario.

These findings affirm the need to integrate culture-independent identification techniques within clinical microbiology laboratory workflow. Implementing MALDI-TOF MS as a method for streamlining the timing for microbial identification and antimicrobial therapy can have significant benefits in terms of clinical outcomes. A meta-analysis study by Yo *et al.* reported that using MALDI-TOF MS for microbial identification in bloodstream infections was associated with a 23% reduction in patient mortality relative to phenotypic methods, with 23-h reduction to microbial identification and 5-h reduction for antimicrobial therapy [21]. MALDI-TOF MS for AST is not as well developed as it is for microbial identification, due to the underdeveloped spectral database for antimicrobial resistance profiles. Further studies investigating the sample preparation techniques and antimicrobial resistance profiles should be performed, as clinical outcomes ultimately depend on the rapid administration of appropriate antimicrobial treatment [23,26]. We reassert that result interpretation must be led by an experienced and competent microbiologist, regardless of the laboratory methods utilized. Laboratories using MALDI-TOF MS should also ensure that they have validated their system with known isolates of organisms they are reporting before accepting the test results with isolates from clinical specimens.

EQA programmes are an integral element to maintain quality laboratory practice and support strong health systems because they provide an objective service of assessment in accordance with the international standards of quality. It is not the role of PT providers to advise laboratories on what technologies they should use, but by presenting the data and allowing laboratories to compare their results to those obtained by other laboratories, they play an important role in quality improvement and may influence laboratory management decisions for implementing new technologies.

### Open access

The authors will make all de-identified laboratory data underlying the findings described in the manuscript fully available upon request.

## Abbreviations

AOR: adjusted odds ratio
AST: antibiotic susceptibility testing
CI: confidence interval
CMPT: Canadian microbiology proficiency testing
DAG: directed acyclic graph
EQA: external quality assessment
MALDI-TOF MS: matrix-assisted laser desorption ionization time-of-flight mass spectrometry
MS: mass spectrometry
PT: proficiency testing
